
JOURNAL INFORMATION
==============================
NLM Title Abbreviation: Wirtschaftsdienst
Journal ID: Wirtschaftsdienst
NLM Journal ID: 101086612

Wirtschaftsdienst (Hamburg, Germany : 1949)

ISSN: 0043-6275

ARTICLE INFORMATION
==============================
PMCID: PMC7678253
PMID: 33250533
DOI: 10.1007/s10273-020-2776-2
Article ID: 2776
Article version: 1
Subjects: Zeitgespräch

Kommt nach der Corona-Krise die Inflation?

Bofinger Peter Bofinger@t-online.de

grid.8379.5 0000 0001 1958 8658 Lehrstuhl für VWL u.a., Universität Würzburg, Sanderring 2, 97070 Würzburg, Deutschland
Electronic publication date: 2020 Nov 20
Print publication date: 2020
Volume: 100
Issue: 11
First page: 825
Last page: 829
Copyright: © Der/die Autor(en) 2020
License: Open Access: Dieser Artikel wird unter der Creative Commons Namensnennung 4.0 International Lizenz (https://creativecommons.org/licenses/by/4.0/deed.de) veröffentlicht.
Open Access wird durch die ZBW — Leibniz-Informationszentrum Wirtschaft gefördert.
License URL: https://creativecommons.org/licenses/by/4.0/

issue-copyright-statement: © The Author(s) 2020

==============================

„Die warnenden Stimmen werden lauter: Immer mehr Finanzexperten in aller Welt befürchten für die absehbare Zeit steigende Inflationsraten in den Industrie- und Schwellenländern." Das klingt aktuell, das Zitat stammt jedoch aus der Frankfurter Allgemeinen Zeitung vom 27. Januar 2011. Wie schon nach der Finanzkrise der Jahre 2008 und 2009 sind jetzt zunehmend Stimmen zu hören, die für die kommenden Jahre mit erheblichen Inflationsgefahren rechnen. Bei den umfangreichen fiskalpolitischen Maßnahmen zur Stützung der von der Corona-Pandemie stark beeinträchtigten Volkswirtschaften und den hoch dosierten Ankaufprogrammen der großen Notenbanken ist es nicht überraschend, dass sich viele Menschen Sorgen um die Stabilität des Geldes machen.

Aber der Rückblick auf die Finanzkrise und auch auf die Eurokrise zeigt, dass sich die damals weitverbreiteten Inflationsszenarien als unzutreffend erwiesen haben. Bei dem noch vergleichsweise kurzen Erfahrungshorizont mit der Corona-Pandemie ist es nicht einfach, die davon möglicherweise ausgehenden inflationären Impulse angemessen zu beurteilen. Die folgenden Einschätzungen sind daher nur als eine grobe Orientierung anzusehen.

## Die bisherigen Erfahrungen

Aus wirtschaftlicher Sicht handelt es sich bei der Corona-Pandemie um einen gleichzeitigen Angebots- und Nachfrageschock. Auf der Angebotsseite stellte sich vor allem am Anfang das Problem unterbrochener globaler Lieferketten sowie einer mangelnden Verfügbarkeit von Arbeitskräften, da diese ihre Kinder betreuen mussten. Länger anhaltende negative Produktivitätseffekte gehen von den Hygienevorschriften aus, die das Einhalten von Abständen und eine laufende Desinfektion vorschreiben. Auf der Nachfrageseite bedeuten die Schließung von Restaurants und Hotels sowie das Verbot von Kongressen und Sportveranstaltungen einen drastischen Schock für Unternehmen und Selbstständige, die Dienstleistungen im Bereich des „sozialen Konsums" anbieten. Zusätzliche negative Effekte gehen von der Kaufzurückhaltung der Konsument*innen aufgrund von Kurzarbeit oder Arbeitslosigkeit sowie der Unsicherheit über die weitere wirtschaftliche Entwicklung aus. Viele Konsument*innen dürften ihre Ausgaben auch aufgrund von Ansteckungsgefahren einschränken. Steigende Infektionszahlen und das Fehlen einer überzeugenden Strategie zur Bekämpfung der Pandemie dürften Unternehmen dazu veranlassen, größere Investitionsvorhaben zumindest aufzuschieben.

Während der Angebotsschock eher preistreibend wirkt, gehen von den Störungen auf der Nachfrageseite eher deflationäre Effekte aus. Bisher sind seit dem Jahresende 2019 keine größeren Auswirkungen der Krise auf die Verbraucherpreise zu erkennen. Während das Preisniveau im Euroraum und in Deutschland im Oktober 2020 saisonbereinigt um rund 0,5 % bzw. 0,4 % unter dem Niveau von Dezember 2019 lag, bewegte es sich in den USA im September mit 0,7 % leicht darüber.

An den Daten für den Euroraum (vgl. Abbildung 1) lässt sich erkennen, dass es bei den Preisen für unverarbeitete Lebensmittel zu Beginn der Krise zu einem deutlichen Preisschub gekommen ist, der sich bis zuletzt nicht zurückgebildet hat. Leicht überdurchschnittlich haben sich auch die verarbeiteten Lebensmittel verteuert. Die Preise für die übrigen Komponenten und der Index insgesamt (ohne Energie) sind mehr oder weniger konstant geblieben. Der Befund spricht zum einen für eine gewisse Preisstarrheit und zum anderen dafür, dass sich die preisdämpfenden Nachfrageeffekte und die preistreibenden Angebotsschocks in etwa die Waage gehalten haben. Eindeutig ist allerdings die Entwicklung bei den Ölpreisen, die sich gegenüber dem Jahresbeginn 2020 nahezu halbiert haben.

## Inflationsentwicklung aus realwirtschaftlicher Sicht

Die Erfahrungen aus den vergangenen Jahrzehnten zeigen, dass die Preisentwicklung in Deutschland, aber auch in anderen fortgeschrittenen Volkswirtschaften, sowohl von den Entwicklungen am Arbeitsmarkt als auch von den Preisschocks am Ölmarkt geprägt gewesen ist.

Die von der Beschäftigungssituation auf die Inflation ausgehenden Impulse lassen sich durch die Phillips-Kurve beschreiben. Sie bildet den Zusammenhang ab, der zwischen der Lohn- und Preisentwicklung auf der einen Seite und Inflationserwartungen und der Situation am Arbeitsmarkt auf der anderen Seite besteht. Im vergangenen Jahrzehnt wurde viel darüber diskutiert, inwieweit sich dieser Zusammenhang abgeschwächt hat. Eine Studie der Deutschen Bundesbank (2016) kommt jedoch zu dem Ergebnis, dass „die Phillips-Kurve nach wie vor als ein wichtiges Instrumentarium der Preisanalyse und -prognose dienen kann“[1].

Vor diesem Hintergrund sollten auf kurze bis mittlere Sicht keine größeren Inflationsgefahren bestehen. Nach der Prognose der Gemeinschaftsdiagnose (2020) vom Oktober 2020 ist damit zu rechnen, dass die Arbeitslosigkeit in allen großen Volkswirtschaften bis 2021 noch merklich ansteigen und auch 2022 um rund einen halben Prozentpunkt über dem Niveau des Jahres 2019 liegen wird. Diese Entwicklung dürfte noch die im Jahr 2022 für die darauffolgenden Jahre abzuschließenden Tariflöhne prägen, sodass von der Lohnseite auf absehbare Zeit nicht mit inflationären Impulsen zu rechnen ist.

Auch über das Jahr 2022 hinaus ist zu erwarten, dass die Arbeitsmarktsituation von den strukturellen Effekten geprägt sein wird, die von der Corona-Pandemie auf das Wirtschaftsgeschehen ausgehen. So ist mit einem großen Digitalisierungsschub in der ganzen Wirtschaft zu rechnen. Eine Studie von Bertschek und Erdsiek (2020) zeigt, wie stark Soloselbstständige die Digitalisierung vorangetrieben haben. Eine Analyse der KfW kommt zu einem ähnlichen Befund für den Mittelstand. In der Industrie dürften die Erfahrungen mit der Corona-Pandemie dazu führen, dass noch stärker als bisher auf Roboter gesetzt wird. Joe Stiglitz (2020) hat das wie folgt auf den Punkt gebracht: „Because machines cannot be infected by the virus, they will look relatively more attractive to employers, particularly in the contracting sectors that use relatively more unskilled labor.“

Negativ auf die Arbeitsmarktperspektiven von weniger qualifizierten Arbeitnehmer*innen dürfte sich auch auswirken, dass in der Post-Corona-Welt mit großer Wahrscheinlichkeit weniger Geschäftsreisen durchgeführt werden. Das bedeutet Arbeitsplatzverluste in den davon abhängigen Dienstleistungsbereichen (Hotels, Fluglinien, Restaurants, Taxis, Bars, Catering). Dienstleistungsbetriebe in den Innenstädten dürften darunter leiden, dass sich dort bei einer zunehmenden Verbreitung von Homeoffice generell weniger Menschen aufhalten werden.

Abbildung 1
**Preisentwicklung der Komponenten des Harmonisierten Verbraucherpreisindex im Euroraum**
Preisindizes 12/2019 = 100

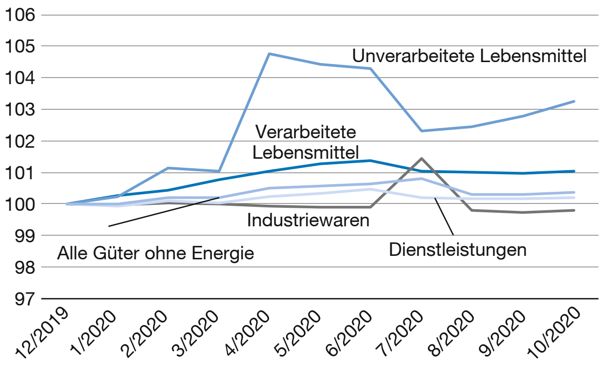

Quelle: Europäische Zentralbank, Statistical Data Warehouse.

Ungünstig für die Perspektiven am Arbeitsmarkt wirken sich zudem die Effekte der Corona-Krise auf das Arbeitsangebot aus. Man spricht dabei nicht mehr von Hysterese-Effekten, sondern von den Narben (*scarring*) die sich aus solchen Schocks für die Berufsaussichten ergeben. Portes (2020) nennt hier folgende Punkte: Generell führt Arbeitslosigkeit zu einer Abschreibung auf das Humankapital. Die beruflichen Fähigkeiten atrophieren und das seelische Befinden leidet, was zu psychischen Krankheiten führen kann. Das beeinträchtige die Einkommens- und Lohnperspektiven in signifikanter Weise. Diese Effekte seien besonders nachteilig für jüngere Arbeitnehmer*innen. Zudem führe der Arbeitsplatzverlust dazu, dass Arbeitskräfte (und Unternehmen) ihr firmenspezifisches Kapital verlieren und dadurch weniger produktiv seien. Schließlich habe die Krise negative Effekte auf die Ausbildung junger Menschen. Schon ein relativ kurzer Verlust an Ausbildungszeit habe erhebliche Konsequenzen für die Entwicklung der beruflichen Fähigkeiten (Burgess and Sievertson 2020).

Die durch die Corona-Pandemie ausgelösten dauerhaften Strukturveränderungen im Bereich der Mobilität dürften sich – zusammen mit der wachsenden Bedeutung erneuerbarer Energien – auch längerfristig dämpfend auf die Preise von Erdöl und Erdgas auswirken. Der Einfluss der Ölpreise auf die Preisentwicklung in Deutschland ist bei einer längerfristigen Perspektive deutlich erkennbar (vgl. Abbildung 2). Man erkennt dabei auch die preistreibenden Effekte der Lohnentwicklung zu Beginn der 1970er Jahre und die dämpfenden Impulse durch die Lohnmoderation, die gegen Ende der 1990er Jahre einsetzte. Insgesamt gesehen ist somit

1 „This paper argues that European inflation behavior is not as puzzling or complex as recent discussions suggest. A simple Phillips curve captures most of the movements in inflation over the twenty years that the Euro has existed“ (Ball und Mazumder, 2019).

Abbildung 2
**Inflationsrate in Deutschland und Veränderung des Ölpreises gegenüber dem Vorjahr**
Inflationsrate in %, Ölpreis: West Texas Intermediate (WTI) in US-$

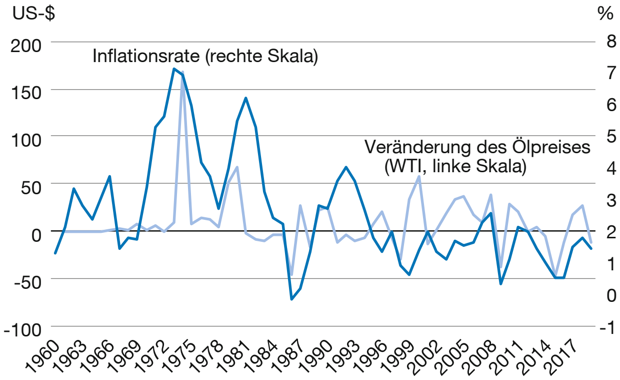

Quellen: Federal Reserve Bank of St. Louis, Deutsche Bundesbank.

Abbildung 3
**Anstieg der Schuldenstandsquoten**
in Prozentpunkten

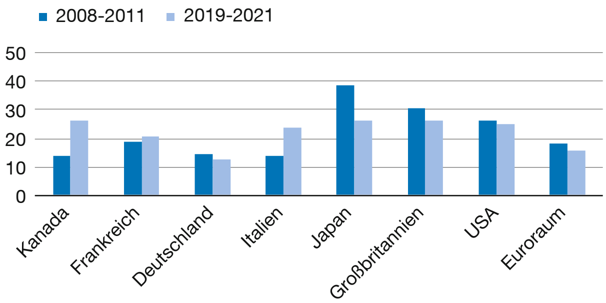

Quelle: Internationaler Währungsfonds, World Economic Outlook Database.

nicht damit zu rechnen, dass es über die Energiepreise mittelfristig zu inflationären Effekten kommen wird. Zum Bild einer durch die Corona-Pandemie mittelfristig geänderten Einstellung zur Mobilität gehören schließlich auch die Effekte auf die Märkte für Gewerbeimmobilien. Der Bedarf an Büroräumen wird ebenso sinken wie die Nachfrage nach Immobilien für Hotels und den stationären Handel. Das wird sich negativ auf die Beschäftigung in der Bauwirtschaft auswirken und somit den Arbeitsmarkt zusätzlich belasten.

Zu alledem wird in der Zeit nach 2021 in vielen Ländern die Notwendigkeit hinzutreten, nach den hohen Defiziten zur Pandemie-Bekämpfung die öffentlichen Haushalte wieder drastisch zu konsolidieren. In Deutschland erfordert die Schuldenbremse, dass die in den Jahren 2020 und 2021 aufgenommenen Schulden in „angemessener Zeit" (Artikel 109 GG) getilgt werden müssen. Bund und Länder stehen also unter dem Zwang, nicht nur einen ausgeglichenen Haushalt, sondern sogar Überschüsse erzielen zu müssen. Wenn an der Schuldenbremse festgehalten wird, was zu befürchten ist, ergeben sich daraus restriktive Nachfrage-Impulse. Wird im Euroraum an den Regeln des Stabilitäts- und Wachstumspakts festgehalten, kommt es zu erheblichen Konsolidierungserfordernissen, vor allem in den von der Pandemie besonders stark betroffenen südeuropäischen Volkswirtschaften.

## Inflationsentwicklung aus monetärer Sicht

Von vielen Betrachter*innen wird die krisenbedingt allgemein stark ansteigende Staatsverschuldung als eine Gefahr für die Geldwertstabilität angesehen. In der Tat ist es in der Geschichte immer wieder vorgekommen, dass dadurch sich selbst verstärkende Prozesse ausgelöst wurden, die in einer Hyperinflation endeten. Entscheidend für die Auswirkungen hoher Budgetdefizite ist die gesamtwirtschaftliche Konstellation. In einer Phase mit großen Engpässen auf der Angebotsseite, wie beispielweise in Deutschland nach dem Ersten Weltkrieg, kann eine staatliche Nachfragestimulierung leicht zur Inflation führen. In einer Situation wie in der Corona-Krise (und der Zeit danach) besteht das Problem jedoch nicht in Angebotsengpässen, sondern vielmehr in einer unzureichenden gesamtwirtschaftlichen Nachfrage, die mit Überkapazitäten einhergeht. Ein abschreckendes Beispiel hierfür ist die große Depression der Jahre 1929 bis 1933, die aufgrund eines unzureichenden fiskalpolitischen Handels von einer massiven Deflation geprägt war.

Obwohl es, wie die Modern Monetary Theory (MMT) zurecht feststellt, für die Fiskalpolitik in großen Volkswirtschaften keine Finanzierungsrestriktion gibt, wird von MMT-Protagonist*innen immer betont, dass die Restriktion der real verfügbaren Ressourcen in einer Volkswirtschaft berücksichtigt werden muss, wenn man vermeiden will, dass es zu Inflation kommt (Tymoigne und Wray 2013).

Dass auch ein starker Anstieg der Staatsverschuldung nicht zu Inflation führen muss, zeigt der Blick auf die Finanzkrise 2008/2009. Damals war der Anstieg der Staatsschuldenquote (Staatsverschuldung in % des nominalen Bruttoinlandsprodukts) in den großen Volkswirtschaften ähnlich hoch ausgefallen, wie das jetzt für die Corona-Krise beispielsweise vom Internationalen Währungsfonds in seinem World Economic Outlook vom Oktober 2020 für die Jahre von 2019 bis 2021 erwartet wird (vgl. Abbildung 3). Wie bereits erwähnt, haben sich die damaligen Inflationsbefürchtungen jedoch als unbegründet erwiesen.

Abbildung 4
**Anleihekäufe der Notenbanken**
in % des Bruttoinlandsprodukts des Vorjahres

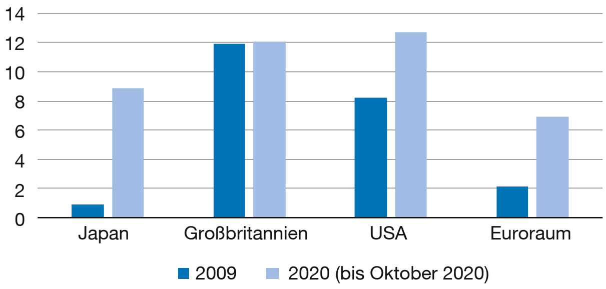

Quelle: Notenbankstatistiken.

Abbildung 5
**Geldmengenwachstum**
in %

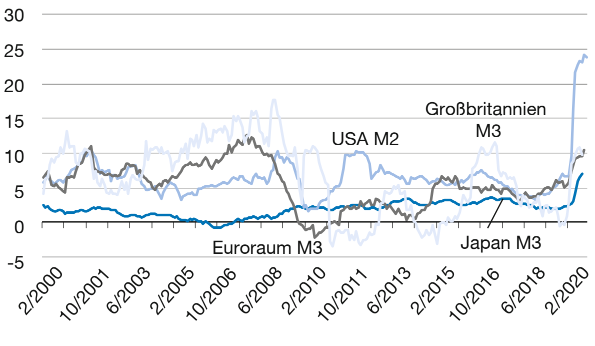

Quelle: Federal Reserve Bank of St. Louis.

Ein deutlicher Unterschied zur Finanzkrise besteht allerdings in der Rolle der Notenbanken. Ende 2019 bis Oktober 2020 haben die Bank von Japan, die Europäische Zentralbank (EZB) und die Federal Reserve deutlich mehr Anleihen angekauft als im gesamten Jahr 2009. Besonders deutlich ist der Unterschied bei der Bank von Japan und der EZB (vgl. Abbildung 4). Dies dürfte eine Erklärung dafür sein, dass 2009 die langfristigen Zinsen für diese Währungen deutlich höher lagen als heute. Die Renditen für US-Dollar-, Pfund-Sterling- und Euroanleihen lagen bei 3,3 %, 3,4 % und 3,8 %. Für Yen-Anleihen beliefen sie sich auf 1,4 %. Aus einer monetaristischen Sichtweise ist von Interesse, welche Auswirkungen sich aus der Bilanzausweitung der Notenbank für die Entwicklung der Geldbestände der Nichtbanken ergeben. In allen vier Währungsräumen ist es durch die Corona-Pandemie zu einer Beschleunigung des Geldmengenwachstums gekommen (vgl. Abbildung 5). Im Fall von Großbritannien und dem Euroraum gehen die Größenordnungen jedoch nicht über Werte hinaus, wie sie auch schon in den 2000er Jahren zu beobachten gewesen waren. In Japan belief sich der Anstieg der Geldmenge M3 auf 7,1 %, was allerdings im historischen Vergleich relativ hoch ist. Mit einer Zuwachsrate von zuletzt 23,9 % fällt das Geldmengenwachstum in den USA demgegenüber völlig aus dem Rahmen.

Diese ungewöhnliche Entwicklung korrespondiert mit einer im historischen Vergleich ebenfalls rekordhohen Sparquote der privaten Haushalte. Diese erreichte im 2. Quartal 2020 in den USA einen Wert von 25,8 %, im 3. Quartal 2020 lag sie mit 15,7 % immer noch auf einem ungewöhnlich hohen Niveau. Zu erklären ist diese Konstellation aus einer Kombination mit extrem hohen staatlichen Transfers an die privaten Haushalte im 2. und 3. Quartal 2020, bei den gleichzeitig stark eingeschränkten Konsummöglichkeiten im 2. Quartal 2020. Die staatlichen Transfers, die vor allem in der Form von sehr großzügigen Arbeitslosengeldzahlungen im Rahmen des CARES Act geleistet wurden, fielen in beiden Quartalen so hoch aus, dass sie die krisenbedingten Ausfälle bei den regulären Haushaltseinkommen bei weitem überkompensierten. Im Ergebnis erzielten die privaten Haushalte im 2. Quartal 2020 einen Einkommensanstieg von 12,6 % gegenüber dem Vorjahresquartal; im 3. Quartal lag der Anstieg immer noch bei 7,7 %. Es ist schwer abzuschätzen, wie sich die durch überzogene staatliche Transfers bei gleichzeitig eingeschränkten Konsummöglichkeiten generierte private Ersparnis auf die Preisentwicklung auswirken wird. Bei Haushalten, deren Konsumverhalten durch eine intertemporale Optimierung gekennzeichnet ist, dürfte das erhöhte Vermögen nur bedingt zu einem Konsumschub führen. Von Haushalten, die ihr Ausgabenverhalten vom laufenden Einkommen abhängig machen, könnte nach dem Ende der Krise ein erhöhter Konsum ausgehen, der eine preistreibende Wirkung entfaltet.

Aus einem solchen Einmaleffekt muss sich keine dauerhafte Inflationsbeschleunigung ergeben. Ganz entscheidend ist dabei, dass die Inflationserwartungen der privaten Haushalte fest verankert bleiben. In diesem Umfeld war die Ankündigung des Notenbankpräsidenten Powell, man werde nach Perioden mit einer Inflationsrate unter 2 % eine Inflationsrate von „moderately above 2 percent for some time" anstreben, nicht unbedingt hilfreich. Für die Erwartungsbildung ist es problematisch, wenn Powell ankündigt, dass sich die Fed dabei nicht an eine bestimmte Formel halten, sondern eine „flexible form of average inflation targeting" verfolgen werde (Powell, 2020).

## Zusammenfassung

Nach der immer noch vergleichsweise kurzen Erfahrung mit den wirtschaftspolitischen und wirtschaftlichen Implikationen der Corona-Pandemie lassen sich deren Auswirkungen auf die Inflationsentwicklung nur grob abschätzen. Aus realwirtschaftlicher Sicht dürften die durch den Schock erhöhte

Arbeitslosigkeit und die negativen Auswirkungen längerfristiger struktureller Veränderungen (geringere Mobilität, erhöhte Digitalisierung, geringere Nachfrage vor allem nach unqualifizierten Arbeitskräften) eine preisdämpfende Wirkung entfalten. Auch bei den Preisen von fossilen Brennstoffen ist sowohl kurzfristig als auch mittelfristig mit keinem steigenden Trend zu rechnen. Ungünstige Perspektiven für Gewerbeimmobilen und der Konsolidierungszwang, dem sich die öffentlichen Haushalte nach dem Abflauen der Pandemie gegenübersehen werden, haben zudem eine eher deflationäre Wirkung.

Aus monetärer Sicht ist eine erhöhte Staatsverschuldung nicht automatisch als inflationär anzusehen. Sofern sie, wie im Fall der Pandemie, dafür sorgt, einem Einbruch der Nachfrage entgegenzuwirken, verhindert sie vielmehr eine Deflation. Von der Größenordnung geht der Anstieg der Schuldenquote in den G7-Staaten nicht über Werte hinaus, die in der Finanzkrise zu verzeichnen waren. Die Finanzierung durch die Notenbanken ist allerdings deutlich stärker ausgefallen als damals, was die im Vergleich zu 2009 wesentlich geringeren Zinssätze für langfristige Anleihen zumindest teilweise erklären kann. Bei der aus monetaristischer Sicht zentralen Entwicklung der Geldbestände von Nichtbanken bewegen sich die Werte für den Euroraum und Großbritannien bisher noch in einer Größenordnung, wie sie schon in den 2000er Jahren beobachtet werden konnte, ohne dass sich daraus Inflationstendenzen ergeben hätten. Auch in Japan ist die Entwicklung noch moderat. Das ist ganz anders in den USA, wo das Geldmengenwachstum weit über die bisher verzeichneten Höchstwerte hinausgeht. Die Ursache hierfür sind ungewöhnlich hohe staatliche Transfers, die ebenfalls eine ungewöhnlich hohe private Ersparnis ermöglichten. Wenn diese nach Abflauen der Krise zu erhöhten Konsumausgaben verwendet würden, könnte dies temporär zu einer Überhitzung führen.

Prof. Dr. Peter Bofinger ist Lehrstuhlinhaber für VWL I, Geld und internationale Wirtschaftsbeziehungen an der Universität Würzburg.


Literatur

Ball, L. M. und S. Mazumder (2019), A Phillips Curve for the Euro Area, NBER Working Paper, 26450, http://www.nber.org/papers/w26450 (3. November 2020).
Bertschek I Erdsiek D Soloselbstständigkeit in der Corona-Krise — Digitalisierung hilft bei der Bewältigung der Krise ZEW-Kurzexpertise 2020 20–08 27
Burgess, S. und H. H. Sievertsen (2020), Schools, skills, and learning: The impact of COVID-19 on education, VoxEU.org, 1. April, https://voxeu.org/article/impact-covid-19-education (3. November 2020).
Deutsche Bundesbank (2016), Die Phillips-Kurve als Instrument der Preisanalyse und Inflationsprognose in Deutschland, Monatsbericht April 2016, 31–46, https://www.bundesbank.de/resource/blob/664886/de60552409f6dd3f4fe614454664d800/L/2016-04-phillips-kurve-data.pdf (3. November 2020).
Frankfurter Allgemeine Zeitung (2011), Banker und Ökonomen befürchten Inflation, 27. Januar, https://www.faz.net/aktuell/wirtschaft/weltwirtschaftsforum-2011/weltweite-preissteigerung-banker-und-oekonomen-befuerchten-inflation-1572787.html (3. November 2020).
Gemeinschaftsdiagnose (2020), Gemeinschaftsdiagnose Herbst 2020: Erholung verliert an Fahrt — Wirtschaft und Politik weiter im Zeichen der Pandemie, Gemeinschaftsdiagnose im Auftrag des Bundesministeriums für Wirtschaft und Energie.
Portes, J. (2020), The lasting scars of the Covid-19 crisis: Channels and impacts, VoxEU.org, 1. Juni, https://voxeu.org/article/lasting-scars-covid-19-crisis (13. November 2020).
Powell, J. H. (2020), New Economic Challenges and the Fed’s Monetary Policy Review. Speech at „Navigating the Decade Ahead: Implications for Monetary Policy“, an economic policy symposium sponsored by the Federal Reserve Bank of Kansas City, Jackson Hole, Wyoming, 27. August, https://www.federalreserve.gov/newsevents/speech/powell20200827a.htm (13. November 2020).
Stiglitz, J. E. (2020), Priorities for the COVID 19 Economy, Project Syndicate, 1. Juli, https://www.project-syndicate.org/commentary/covid-2020-recession-how-to-respond-by-joseph-e-stiglitz-2020-06 (13. November 2020).
Tymoigne, É. und R. L. Wray (2013), Modern Money Theory 101: A Reply to Critics, Levy Economics Institute Working Paper, Nr. 778, November, http://www.levyinstitute.org/pubs/wp_778.pdf (13. November 2020).
Zimmermann, V. (2020), Innovationen in der Corona-Krise: Not macht erfinderisch, KfW-Research, Nr. 295, 13. Juli, https://www.kfw.de/PDF/Download-Center/Konzernthemen/Research/PDF-Dokumente-Fokus-Volkswirtschaft/Fokus-2020/Fokus-Nr.-295-Juli-2020-Innovationen-Corona.pdf (13. November 2020).
